# Weil's Disease

**Published:** 1910-04-23

**Authors:** 


### Medicine.

### WEIL'S DISEASE.

Weil's disease has been recorded from Egypt, and the following account of it from the Wellcome Research Laboratories at Khartoum will interest both those who receive patients coming home from abroad, and those either on shipboard or in practice in the tropics who are likely to meet with the disease. Comparatively rare, but none the less interesting, it has been known under the various synonyms of febrile jaundice, infectious jaundice, epidemic jaundice, Griesinger's disease, bilious typhoid. It is an acute infectious disease characterised by fever, jaundice, enlargement of the spleen and liver, nephritis, and various nervous symptoms. Although occurring in epidemic form, especially in the summer months, it is not a contagious disease.

Its geographical distribution is of interest. In Smyrna it has been more or less endemic since the year 1837, and its recognised presence in Alexandria dates from the year 1870. Griesinger, in Cairo, in 1851 and 1852, called attention to its peculiar features, and differentiated it from yellow fever and bilious remittent fever. At the Kasr-el-Ainy Hospital 132 cases were treated by him, and as a result of various post-mortem examinations he chose the synonym of bilious typhoid, owing to the fact that so many organs were affected. In 1886, during four months, 185 cases were reported from Nauplia, in Greece; and Professor Weil, of Heidelberg, published in 1886 four cases of acute infectious jaundice with swelling of the spleen and nephritis; since then several cases have been recorded from Germany.

Small epidemics have occurred in England, the United States, and China, and more or less doubtful cases have occurred in several towns in Egypt, Greece, Malta, Dalmatia, Syria, and the Ionian Islands. Larrey's "yellow fever" in Cairo in 1800 was probably a form of this disease; and in the *British Medical Journal* of 1898 Colonel Crombie noted amongst the unclassed fevers of hot climates, an outbreak occurring in the Central Provinces of India, which was reported by a native doctor as "yellow fever." Anderson reported an epidemic in Buxar Central Gaol, in India, where 16 cases occurred, and undoubtedly it may occur in other parts of India and other tropical countries, though described under other headings.

The disease generally attacks men between the ages of 20 and 30, having a tendency to affect natives and Greeks, though cases have occurred amongst other Europeans. The disease is not contagious, but one attack seems to confer immunity. It is not confined entirely to the poorest classes, for many cases in an epidemic at Alexandria occurred amongst professional men. Its seasonal incidence seems to reach the maximum between the months of April and October.

According to Sandwith, this disorder is undoubtedly a filth disease, engendered by contamination with sewage and putrid meat. In Alexandria it has become more common since the introduction of a bad system of drainage, and the bulk of the patients affected come from the lowest parts of the town, where the sewers empty into the sea; the suburbs of the town, which are not drained at all, remain apparently unaffected.

The cause is still obscure; there is no direct evidence that the disease is insect-borne. Sandwith is inclined to think that either *Culex fatigans* or *Stegomyia fasciata* is a likely culprit in transmitting it. Taeger and Nauwerk, in Germany, believe that the *Bacillus proteus fluorescens* is the cause of the disease, owing to the fact that this bacillus was isolated from the urine in cases after death had occurred, and because the bacillus was found in ducks and geese which had died in the same locality from a disease in which jaundice was the chief symptom.

The symptoms may be divided into three stages: (1) Primary fever, lasting three to five days; (2) jaundice, about seven to nine days; (3) secondary fever, lasting about seven to nine days. After an incubation period of one or two days the disease is ushered in by a rigor, general pains and vomiting, and a temperature of 102° to 104°. About the third or fourth day jaundice begins with great enlargement of the liver and spleen, together with tenderness. Albuminuria is also present, together with a certain amount of suppression, which may increase till uræmia supervenes. The fever subsides, jaundice and other symptoms disappear, and in the majority of the cases this improvement is followed by a secondary fever.

The nervous symptoms consist for the most part of headache, giddiness, and perhaps delirium, the patient passing more or less into the "typhoid state." Muscular pains, especially in the nape of the neck and the calves of the leg, are intense during the first stage of the disease, and are greatly increased by pressure, this forming a useful diagnostic sign.

The chief complication is that of hyperpyrexia, and it is observed that the convalescence is invariably a protracted one. The mortality occurs chiefly in those above the age of 40, and may vary from 10 per cent. to 60 per cent. The chief pathological conditions present consist of an enlargement of the liver with fatty degeneration and concomitant cloudy swelling, and infiltration of the portal canals with lymphocytes. The spleen is only slightly enlarged. Petechial hæmorrhages are frequently present in the pleura, peri- and endo-cardium, and capillary hæmorrhages are present in the stomach and kidneys. Microscopically the kidneys show a lymphocyte infiltration around the glomeruli.

Formerly there was some difficulty in diagnosing this disease from relapsing fever, but that difficulty no longer exists, owing to the presence in the latter of a spirochæte. It has been suggested that this disease resembles yellow fever, but as the latter has not been known to exist in Egypt infectious jaundice can hardly be confused with it, although some observers state that it is a modified form of yellow fever. Its similarity to acute yellow atrophy must also be borne in mind.

Epidemic jaundice occurred in South Africa during the late war, but Mathias considered that it was a distinct variety of febrile jaundice which frequently follows outbreaks of enteric fever, and not Weil's disease.

The treatment is chiefly symptomatic. Bryce-Orme records what appears to have been a fatal case of infectious jaundice in the Federated Malay States, showing therefore that the disease has probably a wider geographical distribution than is supposed, and is not merely confined to the Mediterranean basin. This observer considered the case to be one of infectious jaundice, the predominating signs and symptoms being intense jaundice, enlarged liver, and albuminuria. There were no indications of the malarial parasite being present in the blood, but there was present a well-marked leucocytosis.

